# Dynamics and Entropy Analysis of a Frictionally Loaded Pendulum

**DOI:** 10.3390/e24091269

**Published:** 2022-09-09

**Authors:** Grzegorz Litak, Marek Borowiec, Krzysztof Da̧bek

**Affiliations:** 1 Department of Automation, Lublin University of Technology, Nadbystrzycka 36, PL-20-618 Lublin, Poland; 2 Department of Applied Mechanics, Lublin University of Technology, Nadbystrzycka 36, PL-20-618 Lublin, Poland

**Keywords:** pendulum, Shannon entropy, non-linear dynamics, friction

## Abstract

We use friction to simultaneously damp and excite a pendulum system. A Froude pendulum attached to a suspension shaft is subjected to a frictional load. We investigate two types of response of the system: regular and chaotic responses, depending on the excitation frequency. A transient chaotic solution was also obtained. We identify the motions using phase portraits, Poincaré maps, and Fourier spectra. Finally, the composite multiscaled entropy was estimated for the specified cases to confirm the preliminary classification.

## 1. Introduction

Pendulum systems are very popular non-linear systems used in scientific and other applications. For example, the payload dynamics in overhead cranes or jib cranes can be understood as examples of pendulums [1,2]. With suitable excitation and damping, such systems are strongly non-linear, exhibiting a variety of solutions, including oscillations and rotations. Possible solutions may be either periodic or non-periodic. The superposition rule, well known in linear systems, is breached leading to non-trivial combinations of solutions, manifest as intermittency and chaotic responses. Simultaneously, particular solutions are difficult to control [3]. To achieve effective control, solution identification is needed. In our work, we further develop the discussion of pendulum systems, including frictional drives.

In technical drive structures, friction is unavoidably present [4]. Friction occurring between the contact surfaces of a rotating shaft and pendulum-like sliding objects may induce vibration.

Froude pendulums subject to frictional loads have been investigated in detail in several studies [5,6,7,8,9,10,11] using different simplified equations. Two coupled Froude pendula were also investigated in [11,12]. However, in our work, the friction torque is variable depending on the motion of the drive shaft. In the next sections, we provide a mathematical model of the proposed system with selected solutions.

In this paper, we discuss the concept of multi-scaled entropy applied to response time series of dynamical systems in the case of a pendulum with a frictional load (previous related studies on mechanical systems include [13,14,15,16]). In the following sections, we define the mathematical model, introduce the entropy approach and discuss simulation results. In the final section, we identify solutions using composite multi-scaled entropy to express the change in the complexity of various solutions.

## 2. Physical and Mathematical Models

**Figure 1:**
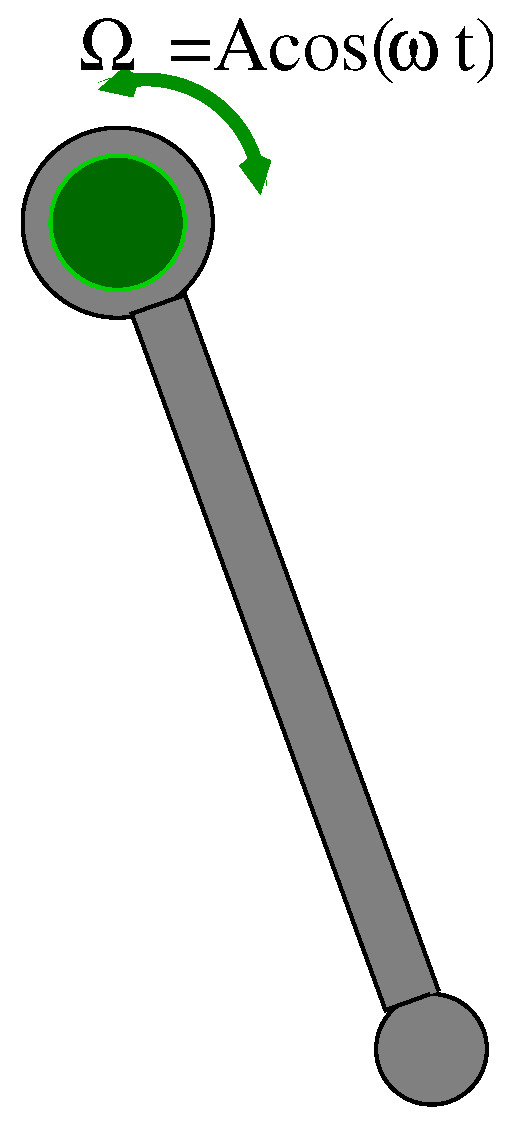
Schematic view of a pendulum subjected to a frictional load of the suspension rotating shaft.

The model examined consists of a physical pendulum suspended on a rotating shaft, driven by harmonical torque through a friction coupling (Figure 1).

The system can be described as a Froude pendulum attached to a rotating suspension shaft which is subjected to a frictional load. The dimensionless equation of motion can be written as:

(1) $$\begin{array}{c} \ddot{\phi }\left(t\right)+\alpha \dot{\phi }\left(t\right)+\gamma \sin\left(\phi \left(t\right)\right)+\mu \;\mathrm{sgn}\left(A\cos\left(\omega t\right)-\dot{\phi }\left(t\right)\right)=0 \end{array}$$

where α is a viscous damping coefficient, μ is the friction force, while γ is the non-linear stiffness-to-mass ratio, and Ω is the variable excitation angular velocity driven harmonically by frequency ω. *A* is the torsional excitation of the internal shaft.

## 3. Multiscaled Entropy

Information entropy was introduced by Shannon [17] to quantify disorder. This concept is directly linked to the Boltzmann formula in the kinetic theory of gases in statistical physics:

(2) $$H=-\eta \sum_{i=1}^{n}p_{i}\log_{2}p_{i},$$

where *H* defines entropy, η is a positive constant depending on the unit of measurement of entropy, and $p_{i}$ is the probability of occurrence of the *i* state. In the kinetic gas theory η coincides with the Boltzmann constant *k*. The above formula can be applied to study regularities in the patterns in various dimensional spaces. In particular, it can quantify dynamical system responses where the corresponding time series can be organized in periodic or non-periodic ways [18]. It is of note that, in non-linear systems, the coarse grain time evolution possesses multiple timescales depending on the sampling time.

In the last 30 years, sample entropy analysis has become increasingly popular for applications in time series analysis [19,20,21,22,23,24].

The method provides a relative level of complexity for a finite length time series [25] containing multiple spatio-temporal correlations. The studied (input) signals can be adopted from measurements or simulations. On the other hand, multi-scale entropy is based on a coarse-graining procedure which provides a number of effective time series, as averages of the original time points within non-overlapping windows of the increasing scale factor τ.

Multi-scale entropy represents a measure of system complexity, which involves an analysis of the response to excitation in terms of time sequence uncertainty.

In the case of a vector character of studied signals, there is a natural phase space (from calculations or reconstruction) representation which defines the *i* (Equation (1)) states’ evolution.

Alternatively, to obtain the multiple resolution of scalar signals, we consider their short finite sequences by introducing ”sampling entropy”, as used by Richman and Moorman (SampEn) [23].

For the time series $X_{i}=\left\{x_{1},x_{2},...,x_{n}\right\}$, with the length of *N* points, one may define so-called *m*-measurement chains of vectors $v\left(i\right)=\left\{x_{i},x_{i+1},\ldots ,x_{i+m-1}\right\}$ and $v\left(j\right)=\left\{x_{j},x_{j+1},\ldots ,x_{j+m-1}\right\}$. Afterwards, one may define the similarity between the vectors v(i) and v(j). The above vectors are similar to each other if their d(i,j)<r, where *r* is a certain tolerance level [26] and d(i,j)=max{|x(i+κ)−x(j+κ)|:0⩽κ⩽m−1} defines the maximum vector element difference of the considered vectors.

The sampling entropy refers here to the information of the v vectors for one scale which is defined by the chain parameter m⩾2. To estimate the complexity of the signal examined at a larger scale, the multi-scale entropy was introduced [21]. This entropy is not calculated by directly comparing v vectors, but by comparing newly created $y^{\left(\tau \right)}$ vectors at a so-called scale factor τ. The vectors are created from coarse-grained time series as follows:

(3) $$y_{j}^{\left(\tau \right)}=\frac{1}{\tau }\sum_{i=(j-1)\tau +1}^{i=j\tau }x_{i},\;\;\;\;1⩽j⩽N/\tau ,$$

where τ=1,2,3.... In accordance with the above formula, $y_{j}^{\left(\tau =1\right)}=x_{i}$. For the non-zero τ, the analysed series $X_{i}$ is a part of the average chain N/τ, where each one has the length of τ. The average value of the calculated chains according to Equation (3) now constitutes a new coarse-grained time series $y^{\left(\tau \right)}$.

**Figure 2:**
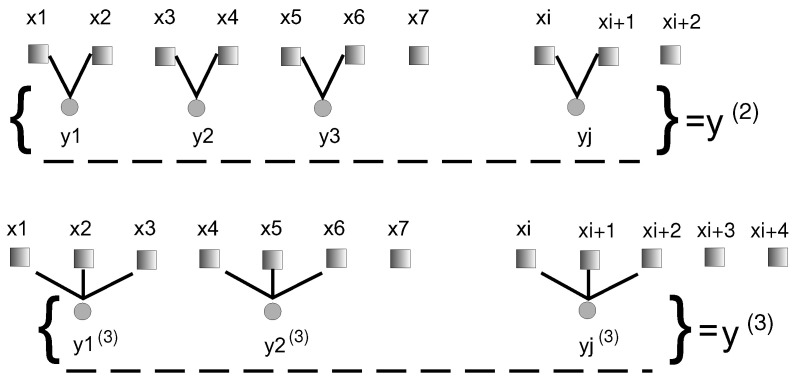
Diagram of coarse-graining procedure for the scale factor τ=2 and τ=3 in MSE.

In Figure 2, a coarse-graining procedure is presented for τ=2 and τ=3. The averaging procedure introduces the smoothing of newly created $y^{\left(\tau \right)}$ vectors, based on the original time series $X_{i}$.

The multi-scale entropy for scales *m* (chain representation) and τ (averaging scale) from the coarse-grained vector $y^{\left(\tau \right)}$ is defined by the following formula:

(4) $$\mathrm{MSE}\left(x,\tau ,m,r\right)=\mathrm{SampEn}\left(y^{\left(\tau \right)},m,r\right).$$

Whereas, $\mathrm{SampEn}\left(y^{\left(\tau \right)},m,r\right)$ in Equation (4) is defined as follows:

(5) $$\mathrm{SampEn}\left(y^{\left(\tau \right)},m,r\right)=\ln\left(\frac{N_{n}}{N_{d}}\right).$$

Values $N_{d}$ and $N_{n}$ are calculated from the previously prepared coarse-grained data $y^{\left(\tau \right)}$ by the following algorithm:

(6) Nd=Nn=1,if|y(τ)(i)−y(τ)(j)|<r&|y(τ)(i+1)−y(τ)(j+1)|<r Nn=Nn+1,if|y(τ)(i+2)−y(τ)(j+2)|<r Nd=Nd+1.

The result of Equation (4) is the probability of occurrence in the next points of the time chain series with lengths *m*, which are similar to each other within the tolerance *r*. In the literature, recommended values of the parameters *m* and *r* are provided [27] for use in calculations of multi-scale entropy. In the case study, these parameters are selected according to the sampling procedure and the length of the time series.

For our analysis of the time series, m=2 was accepted, whereas the tolerance of probability $r=0.2\;\sigma_{x}$, where $\sigma_{x}$ is a standard deviation of the original time series of the $X_{i}$ vector. For introduced values, the whole scope of the scale factor parameter τ, at a level of tolerance *r*, is established as constant [28].

The estimation of complexity of the signal in terms of the multi-scale entropy MSE may be affected by error, depending on the multiple choice of the neighboring points in the averaging procedure. This depends on the accepted length of the index of scale factor τ [29]. Therefore, the authors of [29] introduced a modified form of entropy, which eliminates the error, termed composite multi-scale entropy (CMSE). If the calculations are conducted for the parameter τ∈(0–20), then the error is small and both results, the MSE and the CMSE, are accepted as consistent.

The composite multi-scale entropy (CMSE), in comparison with the diagram presented in Figure 2 and Equation (3), describing the coarse-graining process, coincides only in the first grained series $y_{j}^{\left(\tau =1\right)}=x_{j}$, where k=1. To calculate CMSE, the all coarse-grained time series are included into the following form:

(7) $$y_{k,j}^{\left(\tau \right)}=\frac{1}{\tau }\sum_{i=(j-1)\tau +k}^{i=j\tau +k-1}x_{i},\;\;\;\;1⩽j⩽N/\tau ,\;\;\;\;1⩽k⩽\tau .$$

Then the formula which defines the composite multi-scale entropy CMSE takes the form:

(8) $$\mathrm{CMSE}\left(x,\tau ,m,r\right)=\frac{1}{\tau }\sum_{k=1}^{\tau }\mathrm{SampEn}\left(y_{k}^{\left(\tau \right)},m,r\right).$$

**Figure 3:**
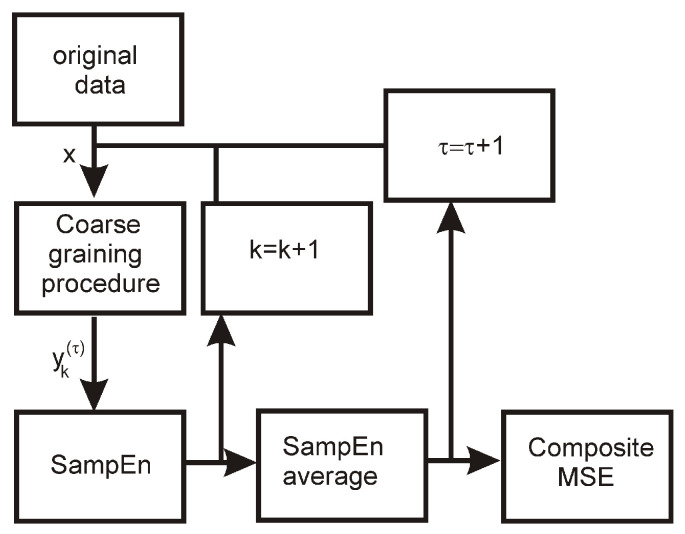
The algorithm of composite multi-scale entropy. *x* is the original time series element, *k* denotes the consecutive point of the time series, τ is the averaging scale, and $y_{k}^{\left(\tau \right)}$ is the element of the effective (scaled) time series.

The algorithm of the composite multi-scale entropy is presented in Figure 3.

The numerical procedure of entropy estimation (from experiments and mathematical models), in the forms presented above, have been applied to physical phenomena in the field of physiology [19,20,21,22,23], medicine [27,30], mechanics [13,14,16,18], and thermodynamics [15], etc. In the present paper, the CMSE is applied to signals of a pendulum system with a friction effect. The results of simulations and corresponding analyses are presented in the next section.

## 4. Results and Discussion

The investigated system is based on a pendulum. There are different responses that can be expected, including rotations and oscillations, as well as chaotic or regular movements. To obtain various types of pendulum behavior, we assumed the following set of system parameters:

(9) α=0.0,γ=1,A=5.2,μ=0.5.

It should be noted that α=0 determines a nodal linear damping but that the fairly large amplitude *A* provides damping and excitation of the same level through a friction phenomenon.

Note that a friction phenomenon occurs in two ways with addition and removal of mechanical energy, depending on the relative motions of the pendulum and the shaft.

**Figure 4:**
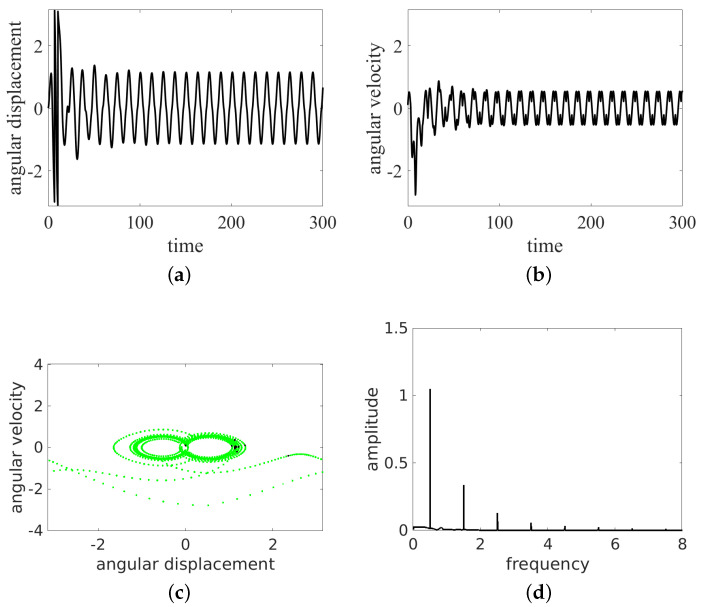
Results for ω=0.5: oscillation. Time series (**a**,**b**), phase portrait (by green dots) and Poincaré points (by black dots) (**c**) and FFT (**d**).

**Figure 5:**
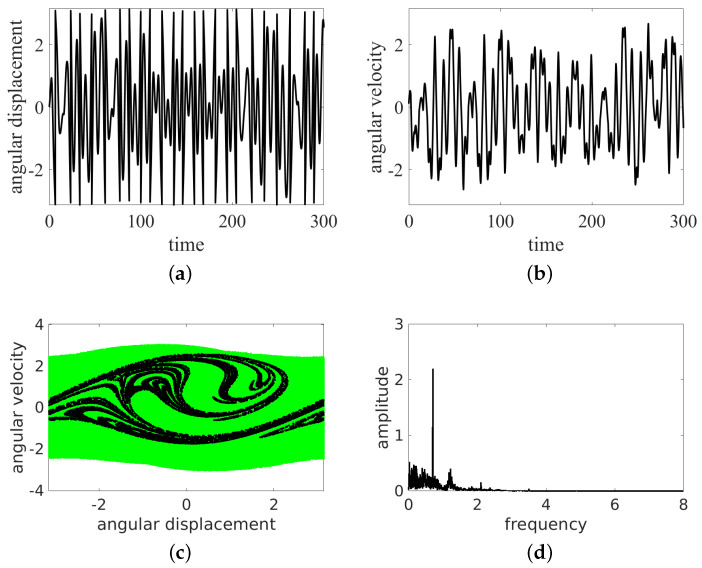
Results for ω=0.7: rotation and oscillation. Time series (**a**,**b**), phase portrait (by green dots) and Poincaré points (by black dots) (**c**) and FFT (**d**).

**Figure 6:**
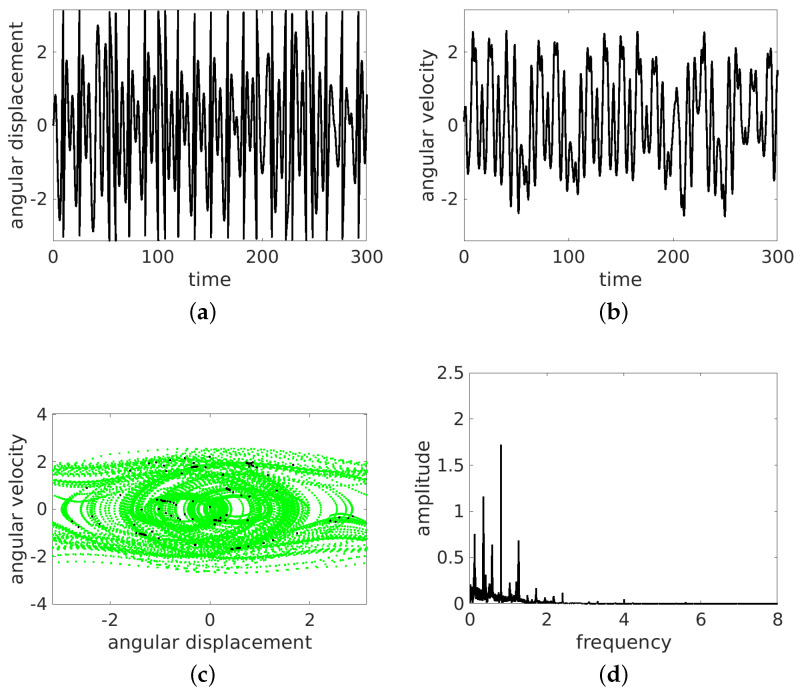
Results for ω=0.8: rotation and oscillation. Time series (**a**,**b**), phase portrait (by green dots) and Poincaré points (by black dots) (**c**) and FFT (**d**).

**Figure 7:**
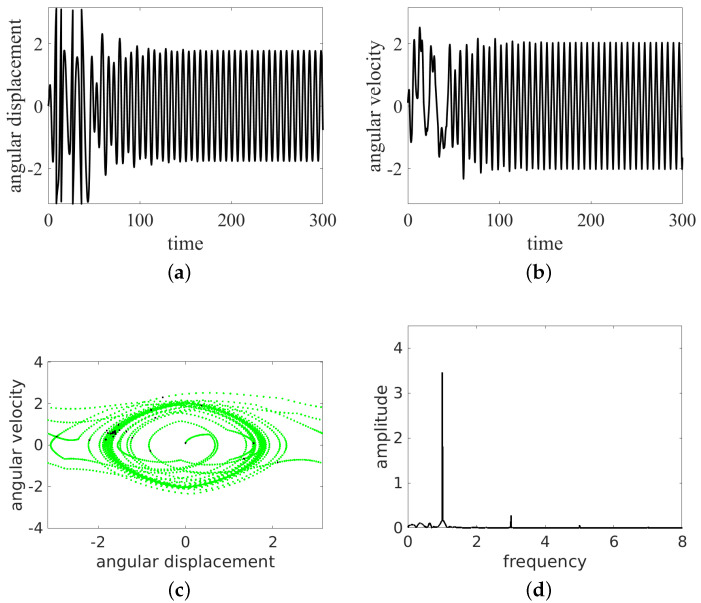
Results for ω=1.0: oscillation. Time series (**a**,**b**), phase portrait (by green dots) and Poincaré points (by black dots) (**c**) and FFT (**d**).

**Figure 8:**
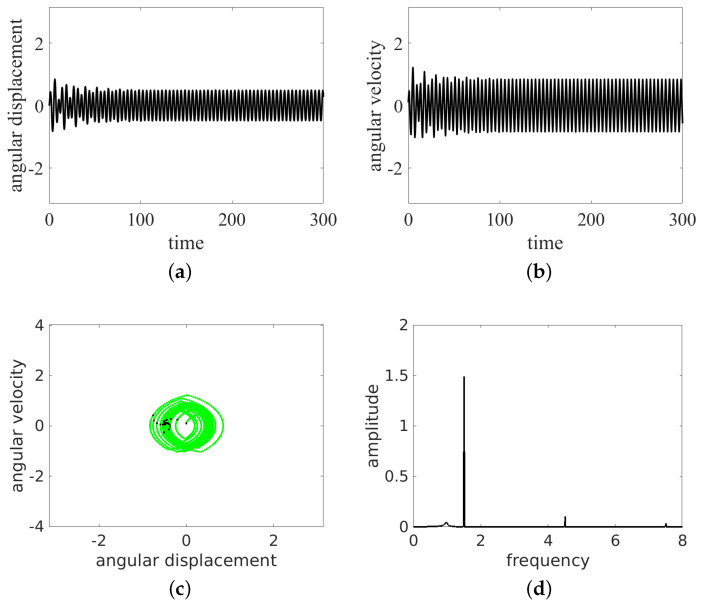
Results for ω=1.5: oscillation. Time series (**a**,**b**), phase portrait (by green dots) and Poincaré points (by black dots) (**c**) and FFT (**d**).

In the present simulations, we changed the frequency ω= 0.5, 0.7, 0.8, 1.0, and 1.5. The results of the time histories (angular displacement and angular velocity), the phase portraits, and the Fourier spectra of angular velocity are presented in Figure 4, Figure 5, Figure 6, Figure 7 and Figure 8. They are marked with subfigure labels of (a), (b), (c), and (d), respectively. In the Runge–Kutta (of the fourth order) integration of Equation (1), we used a single set of the initial conditions $\left(\phi_{0},{\dot{\phi }}_{0}\right)=\left(0.0,0.1\right)$; the integration time step was variable, with sampling of results δt fixed to 2π/ω/100. Such a sampling is sufficient for solution identification, taking into account the timescales in the system.

The results show that the pendulum can move in periodic (Figure 4 and Figure 6, Figure 7 and Figure 8) and non-periodic (Figure 5) ways. Note also that the non-periodic transient are always present in the initial stage of motion. Note that the angular displacement was transformed to a modulo [−π,π] function to express the cylindrical angular variables. In the lower ω (ω= 0.5, 0.7, 0.8, 1.0) simulation, the results start from simulations, while for ω=1.5, the initial transient motion is the oscillation mode. Note that the phase portraits with Poincaré points (Figure 4, Figure 5, Figure 6, Figure 7 and Figure 8c) are smeared because of the inclusion of transients; however, it is possible to distinguish the periodic from chaotic tendencies. In the case of the non-periodic (chaotic − ω=0.7) solution, the Poincaré points are formed into a strange attractor. In the periodic cases (ω= 0.5, 0.8, 1.0, 1.5), the organization of Poincaré points are attracted by singular points (the most common regular periodic attractor). Simultaneously, the phase portraits form closed loops. In the case of the chaotic attractor, the loops are lines which are looped incorrectly and can be considered open.

In the next analysis, the Fourier spectra of the particular cases are compared. The FTT was calculated using angular velocity histories to avoid results manipulation by the modulo function in the results of the angular displacement variable.

As expected, the cases ω= 0.5, 0.7, 1.0, 1.5 have strong discrete frequency lines in their Fourier spectra, with various harmonics, including superharmonics in the case of ω= 0.5, 1.0, 1.5, and subharmonics in the case of ω= 0.8. In the linear vertical scale the relative heights of the main peaks are clearly visible. Note that the excitation frequency is always present in the response spectrum as expected.

All the regular response cases (ω= 0.5, 0.8, 1.0, 1.5) are slightly disturbed by the transient initial phases of responses and show some low level of the continuous spectrum. On the other hand, the non-periodic solution (at about ω=0.7) shows a rather continuous high level frequency spectrum. The discrete line at ω/3 is shown to make clear that this is the leading subharmonic trend in generally non-periodic behavior.

**Figure 9:**
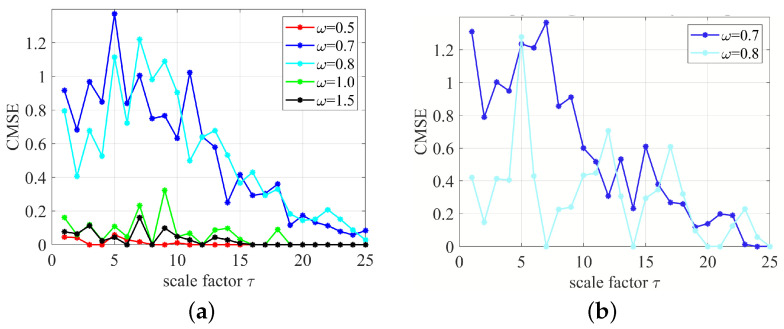
(**a**) Composite multiscale entropy CMSE results, corresponding to the signal of velocity for the whole signals (25,000 velocity points selected four times per excitation period). Note that the regular solutions (ω= 0.5, 1.0, and 1.5) have a much lower level of entropy reducing to almost zero at several τ, while chaotic (ω=0.7) and transient-chaotic (ω=0.8) response entropies are fairly large for almost the whole scope of τ. (**b**) CMSE calculated for the last 120 velocity points for ω= 0.7 and 0.8.

Finally, we performed an analysis of multiscale entropy. The results are presented in Figure 9. For this purpose, we examined the stationary intervals of the cases studied.

The results of composite multi-scale entropy calculations are provided for similarity factor r=0.2σ to show the different CMSE values by increasing the scale factor τ (see Figure 9a). The higher values of CMSE reflect the greater complexity of the analysed signal. It can be clearly seen that the periodic signals are characterized by lower levels of entropy as a function of the scale factor τ. For modulated multiharmonic signals (ω= 0.5), the entropy can oscillate, reaching small values for selected τ expressed in the sampling periods. On the other hand, the chaotic solution has a large entropy level for the whole range of τ. Interestingly, the case ω= 0.8 shows long transient chaos due to the short length of the time series being investigated. Therefore, the cases ω= 0.7 and 0.8 are similar in terms of entropy. For enhanced clarity, we show (Figure 9b) the CMSE results for stationary solutions (after transient cut-off). Note that the case ω= 0.8 shows an oscillatory character with touching CMSE = 0, while the case ω= 0.7 manifests a quasi hyperbolic shape, as for a stochastic white noise signal [22].

## 5. Conclusions

We examined a non-linear pendulum system with multiple solutions. Simulations performed exhibited single and multiple excitation periodic responses, as well as chaotic behavior for the chosen parameters. For practical reasons (control as well as vibration mitigation), it is necessary to identify the type of solutions. Our investigations show that CMSE is a valuable tool in assessing the periodicity and non-periodicity of the investigated system responses for relatively short time series with transient states.

In the next stage, we plan to investigate the signaled bifurcations in more detail.

## Data Availability

Data are contained within the article.
